# Has equity in government subsidy on healthcare improved in China? Evidence from the China’s National Health Services Survey

**DOI:** 10.1186/s12939-017-0516-z

**Published:** 2017-01-10

**Authors:** Lei Si, Mingsheng Chen, Andrew J. Palmer

**Affiliations:** 1 Menzies Institute for Medical Research, University of Tasmania, Medical Science 1 Building, 17 Liverpool St (Private Bag 23), Hobart, TAS 7000 Australia; 2 School of Health Administration, Anhui Medical University, Meishan Road 81, Hefei, 230032 Anhui Province People’s Republic of China; 3 School of Health Policy & Management, Nanjing Medical University, 211166 Nanjing, China

**Keywords:** Benefit incidence analysis, Equity, Government health subsidy, Healthcare

## Abstract

**Background:**

Monitoring the equity of government healthcare subsidies (GHS) is critical for evaluating the performance of health policy decisions. China’s low-income population encounters barriers in accessing benefits from GHS. This paper focuses on the distribution of China’s healthcare subsidies among different socio-economic populations and the factors that affect their equitable distribution. It examines the characteristics of equitable access to benefits in a province of northeastern China, comparing the equity performance between urban and rural areas.

**Methods:**

Benefit incidence analysis was applied to GHS data from two rounds of China’s National Health Services Survey (2003 and 2008, *N* = 27,239) in Heilongjiang province, reflecting the information in 2002 and 2007 respectively. Concentration index (CI) was used to evaluate the absolute equity of GHSs in outpatient and inpatient healthcare services. A negative CI indicates disproportionate concentration of GHSs among the poor, while a positive CI indicates the GHS is pro-rich, a CI of zero indicates perfect equity. In addition, Kakwani index (KI) was used to evaluate the progressivity of GHSs. A positive KI denotes the GHS is regressive, while a negative value denotes the GHS is progressive.

**Results:**

CIs for inpatient care in urban and rural residents were 0.2036 and 0.4497 respectively in 2002, and those in 2007 were 0.4433 and 0.5375. Likewise, CIs for outpatient care are positive in both regions in 2002 and 2007, indicating that both inpatient and outpatient GHSs were pro-rich in both survey periods irrespective of region. In addition, KIs for inpatient services were −0.3769 (urban) and 0.0576 (rural) in 2002 and those in 2007 were 0.0280 and 0.1868. KIs for outpatient service were -0.4278 (urban) and -0.1257 (rural) in 2002, those in 2007 were −0.2572 and −0.1501, indicating that equity was improved in GHS in outpatient care in both regions but not in inpatient services.

**Conclusions:**

The benefit distribution of government healthcare subsidies has been strongly influenced by China’s health insurance schemes. Their compensation policies and benefit packages need reform to improve the benefit equity between outpatient and inpatient care both in urban and rural areas.

## Background

Achieving universal health coverage (UHC), which aims to ensure all people have equal access to healthcare services according to need without the risk of financial crisis [1], is a main goal for health reform in China. In reality, patients may not use healthcare services due to high costs, therefore government healthcare subsidy (GHS) plays a critical role in lowing costs for healthcare, especially for the poor. Given a limited budget, the GHS should be provided in an efficient and equitably way. Low-income and disadvantaged populations are prone to catastrophic payment due to high healthcare out-of-pocket (OOP) costs, therefore should be placed in the top priorities to receive the government subsidies. However, recent evidence has shown that it is often the patients in the upper income groups who reap the largest benefits from public spending programs in health sector [2, 3].

The price of medical services and medications is controlled below the market value in China in order to improve affordability of these services [4, 5]. On the other hand, the government compensates hospitals for deficits between low healthcare prices and high hospital costs [6]. Government funding accounted for about 60% of revenues in public hospitals at the beginning of the 1980s, but it had shrunk to 15.69% in 2002 [6]. Since 2003, China has been gradually launching health sector reforms and has been implementing a specific policy package. The New Rural Cooperative Medical Scheme (NCMS) was a 2003 initiative to rebuild rural health insurance after the collapse of the Rural Cooperative Medical Scheme (CMS) in the late 1980s. In Chinese cities in 2007, a new type of urban insurance, the Urban Resident Basic Medical Insurance (URBMI), was piloted to expand the coverage of the Urban Workers Basic Medical Insurance (UWBMI), which only covers the workers in formal sectors such as state-owned and collective enterprises. Citizens who did not have a job, such as children, the aged or students, were allowed to be covered in the URBMI. This basket of policies made a significant change to the GHS, with government spending as a proportion of health expenditure increasing from 15.69% in 2002 to 22.31% in 2007 [6].

Despite GHS increasing by approximately 7% of total health expenditure from 2002 to 2007, there is no evidence as to whether the distribution of GHS is equitable. Moreover, the changes and variations in the equitable distribution of China’s GHS over the years have not been adequately studied. Therefore, the aims of this study were two-fold: first, to investigate whether the GHS is equitably distributed; and second, to compare the equity of GHSs in different healthcare services between urban and rural residents from 2002 to 2007.

## Methods

### Data sources

Data sources comprised two rounds of China’s National Health Services Survey (NHSS) in Heilongjiang province. The two rounds were conducted in 2003 and 2008 in the sampling areas, reflecting the status in 2002 and 2007 respectively. Heilongjiang province, located in the northeast of China, is a middle-income province in terms of *per capita* GDP. Adopting a multi-stage stratified random sampling method, 13 out of 120 cities and counties in Heilongjiang province were randomly selected in the survey. In every city or county, eight communities or villages were selected according to regional economic level and geographic distribution. From these communities or villages, 33 households were then randomly selected. Each member of the selected families was interviewed by the trained data collectors. Finally, 11,572 individuals in 2003 and 15,817 individuals in 2008 were effectively collected in the survey. This study was approved by the Academic Research Ethics Committee of Nanjing Medical University. Informed consent was obtained from all participants.

The survey contains extensive information about household socio-economic and demographic characteristics, including household expenditure, urban–rural classification, number of family members, gender, age, education attainment and working status of household members, household goods and consumption. Household expenditure per equivalent adult was used as the measure of living standard in our study. The equivalent adults were calculated using the following formula:

$$ AE={\left(A+0.5K\right)}^{0.75} $$

where *A* is the number of adults in the household and *K* represents the number of children (0–14 years) [7].

*Per capita* GHS was computed from two sources. One was from the survey, recording the information from the interviewees on healthcare utilization, such as outpatient visit, length of hospital stay, level of healthcare facility (municipal hospital, county hospital, hospital of traditional Chinese medicine, township hospital, community health center, etc.). The number of inpatient days was reported for a 12-month recall period and outpatient visits for the previous two weeks. The other source of data, which was collected from the local health financial yearbook, was used to calculate the cost of government subsidies, outpatient visits, inpatient days, and revenue at each level of healthcare facility. Since the subsidies were not disaggregated by service type within healthcare facilities, we computed the ratio of outpatient to inpatient revenue as the proportion of subsidies between outpatient and inpatient services. Unit subsidy at the level of healthcare facility was calculated by dividing the total service-specific subsidies at different levels of facilities by the sum of outpatient visits and the number of inpatient days. The subsidy for each individual was the quantity of healthcare utilization multiplied by the unit subsidy at each level of facility.

### Data analysis

Benefit Incidence Analysis (BIA) with concentration index (CI) and Kakwani index (KI) was used to measure the extent of equity in this study. BIA provides an important insight of the assessment of distributional impacts of public spending by combining data on household level with hospital-related costs. It evaluates the distribution of government subsidies for healthcare among different groups in the population, and in particular, among the different income groups [8].

**Fig. 1:**
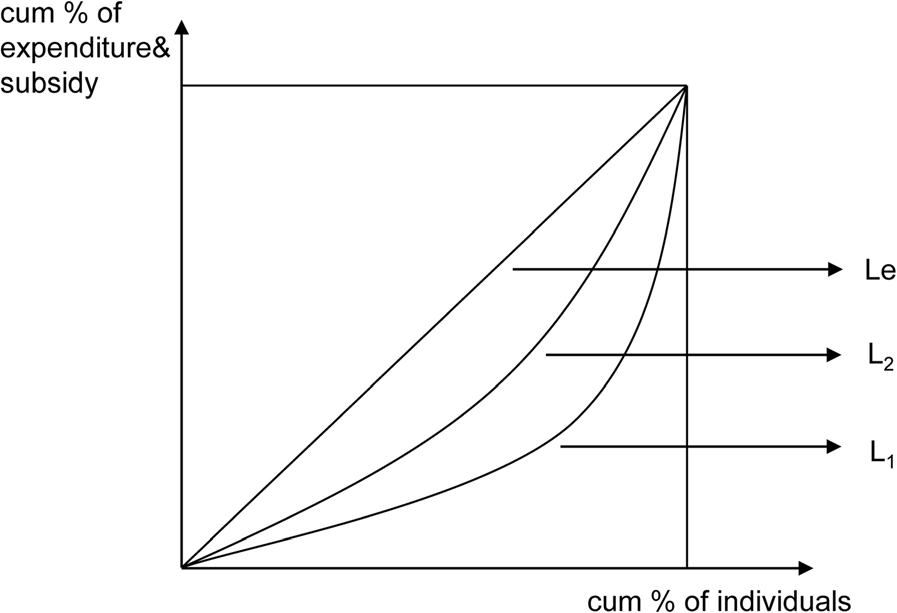
Conceptual concentration curve for government subsidies on healthcare and income. The figure displays the conceptual concentration curve for government subsidies on healthcare and per capita income. The concentration curve plots the cumulative percentage of health subsidy (*y-axis*) against the cumulative percentage of the population (*x-axis*), ranked by living standard from the poorest to the richest. It is measured by CI, as twice the area between the concentration curve, L1, and the line of equality (the 45° line running from the bottom-left corner to the top-right, Le). The Lorenz curve (L_2_) represents the relationship between the cumulative percentage of per capita income and the cumulative percentage of the population, which is measured by the Gini coefficient

The concentration curve and its related CI was used to evaluate the degree of income-related inequity in the distribution of a health variable. Figure 1 displays the conceptual concentration curve for government subsidy on healthcare across individuals and their income. The y-axis is the cumulative percentage of the health subsidy and the x-axis represents the cumulative percentage of the population, ranked by living standards, beginning with the poorest and ending with the richest. The CI is calculated as twice the area between the concentration curve, L_1_, and the line of equality (L_e_, the 45-degree line running from the bottom-left corner to the top-right). The CI compares a concentration curve with the 45-degree line. If the concentration curve lies above the 45-degree line, the distribution is determined to be pro-poor, and if below, the distribution is pro-rich. Pro-poor indicated that the subsidies close the absolute gap in welfare between the rich and poor, while pro-rich indicates an increasing gap [9]. These are called strongly progressive and strongly regressive, respectively. A negative value of CI, where the concentration curve lies above the 45-degree line, indicates disproportionate concentration of government health subsidies among the poor. The further the curve is above the line, the more concentrated the subsidy is amongst the poor and the higher the value of the CI, and vice versa.

However, the CI is only appropriate if the distributional goal is to close the absolute gap in welfare between the rich and poor and it does not take into account the ability-to-pay (ATP) on the relative scale, i.e. progressivity of GHS is not measured by CI. The KI is the most widely used measure of progressivity in health care financing [10]. The KI evaluates the relative gap of subsidy between the poor and rich, and is defined as twice the area between the Lorenz curve (L_2_) and the concentration curve for GHS (Fig. 1). The L_2_ represents the relationship between the cumulative percentage of living standard and the cumulative percentage of the population. If the objective is to close the relative gap, the subsidy should be inequality-reducing (weakly progressive), which requires that the share of subsidy received by the poor exceeds its share of living standard. In this case, the subsidy concentration curve lies above the L_2_ [9]. With the L_2_, the Gini coefficient of living standard, defined as twice the area between the L_2_ and the 45-degree line, can be calculated. Furthermore, the KI can be calculated from the CI and Gini coefficient as the difference between the CI and Gini coefficient.

Therefore:

$$ {\uppi}_k=2{\displaystyle \underset{0}{\overset{1}{\int }}}\left[{L}_2-{L}_1\right]dp $$

$$ {\uppi}_k=2{\displaystyle \underset{0}{\overset{1}{\int }}}\left[{L}_e-{L}_1\right]dp-2{\displaystyle \underset{0}{\overset{1}{\int }}}\left[{L}_e-{L}_2\right]dp $$

$$ {\uppi}_k=C-G $$

where *C* is the concentration index for government subsidy on healthcare, and *G* refers to the Gini coefficient. The KI, π_*k*_, is calculated by the difference between the CI and the Gini coefficient, denoting the degree of progressivity of government health subsidy. Despite that there are several methods for computing the variance of CI [11], convenient regression method has been used in our study. Given the relationship between variance and convenient regression, an equivalent estimate of the CI can be obtained from an ordinary least squares regression of a transformation of GHS of interest on the fractional rank in the living standards distribution [10]. In the context of GHS, it should be inequity-reducing, i.e., the GHS concentration curve dominates the Lorenz curve. On the contrary, if the GHS concentration curve is dominated by the Lorenz curve, the GHS is not equitably distributed. Put it in another way, a positive value of KI (π_*k*_ > 0) indicates the GHS is pro-rich, while a negative value (π_*k*_ < 0) implies the GHS is pro-poor. In the case of proportionality, the KI equals to 0 (π_*k*_ = 0). Moreover, some studies using CI method for other geographic areas within China help to evaluate and understand the extent of health care inequity [12, 13].

In addition, a dominance test was performed. To establish whether the subsidy on healthcare reduces inequity, in the sense that lower income individuals receive a greater share of subsidy than the wealthy, as compared to their living standards, a test was conducted to assess whether the concentration curve dominates (lies above) the L_2_ of household expenditure. For the dominance tests, standard errors of the ordinates of curves and of differences in ordinates were computed, allowing for dependence between curves where appropriate [14]. A multiple comparison approach to testing was adopted, with the null defined as curves being indistinguishable [15].

## Results

Table 1 summarizes the demographic and socioeconomic characteristics in each income quintile of the study population. It was found that the insurance coverage in rural areas was lower than that in cities in 2002, while the coverage had become much higher than in the urban areas by 2007. In 2002, a greater proportion of urban residents received GHS on both outpatient and inpatient care than rural residents. In addition, the OOP payment for inpatient care in urban residents was higher than that in rural residents, but not for outpatient care. In 2007, there were still a larger proportion of urban residents receiving GHS on outpatient care, but not in inpatient care. The OOPs, however, were all higher in urban residents in both inpatient and outpatient care.

**Table 1:** Descriptive statistics of sampling data and socioeconomic characteristics by per capita expenditure quintiles

Year	Income quintiles	per capita expenditure ^a,b^		Insurance rate (%)		% of outpatients receiving imbursement		% of inpatients receiving imbursement		Outpatient OOP ^a,b^		Inpatient OOP ^a,b^	
		urban	rural	urban	rural	urban	rural	urban	rural	urban	rural	urban	rural
2002	Q_1_	4059.86	3648.69	18.89	5.04	17.14	2.78	9.52	18.75	535.21	232.73	2339.55	575.42
	Q_2_	6856.98	5650.99	30.76	7.85	21.74	7.32	23.81	9.09	257.08	472.83	2317.79	1565.06
	Q_3_	10359.93	7572.59	36.42	9.14	29.41	7.46	44.74	11.11	314.89	540.44	3050.69	1857.69
	Q_4_	16039.95	10004.23	48.61	12.87	24.68	5.19	56.52	6.38	470.98	808.41	3407.48	2764.67
	Q_5_	41451.14	16793.03	52.96	13.92	25.71	3.85	54.00	4.08	542.62	1065.09	5779.82	2776.86
	total	15747.00	8731.38	37.51	9.75	24.66	5.35	43.75	8.00	423.76	700.00	4243.90	2283.90
2007	Q_1_	6660.52	6648.07	26.79	84.96	11.76	40.00	22.22	75.00	271.82	255.04	3315.79	1857.33
	Q_2_	9991.07	9728.19	36.86	89.19	14.67	19.47	31.03	72.73	353.45	247.77	2763.45	2283.42
	Q_3_	13350.52	12281.31	45.07	89.66	40.30	25.66	43.59	77.11	415.12	295.48	3120.64	2591.91
	Q_4_	18358.27	15648.48	49.59	89.23	30.43	26.61	49.18	72.90	572.21	217.23	3488.25	2346.19
	Q_5_	37131.60	26416.39	69.74	93.64	49.51	25.19	56.32	67.23	533.02	322.15	10074.57	4593.18
	total	17094.69	14145.80	45.96	89.34	31.61	26.43	46.58	71.62	452.49	269.54	5761.21	3213.14

Data source: author’s calculations of household surveys

^a^All expenditures are presented in CNY

^b^All 2002 nominal prices have been adjusted to real prices in 2007 according to China’s Consumer Price Index (CPI)

Quintile shares of the subsidies on outpatient and inpatient care are shown in Table 2. In addition, total subsidies across urban and rural populations for 2002 and 2007 were also summarized. The benefit incidence of government subsidy was presented using benefit distribution, CI and KI. In 2002 and 2007, the values of CI were all positive, suggesting that a greater proportion of the subsidy was allocated to the rich than to the poor in both outpatient and inpatient services. In the same region, in a particular year, the value of CI for outpatient care is smaller than for inpatient service. The result indicates that compared to outpatient care, pro-rich bias is obviously found in the subsidy distribution of inpatient care among all the regions examined.

**Table 2:** Distribution of government healthcare subsidy (GHS) by income quintiles, concentration index (CI), and Kakwani index (KI)

Year	Area	Income quintiles	Living standard	Outpatient GHS	Inpatient GHS	Total GHS
2002	Urban (A)	poorest 20%	4.93%	13.75%	11.33%	13.11%
		2nd poorest	8.51%	14.58%	14.43%	14.54%
		3rd	12.69%	23.26%	24.89%	23.70%
		2nd richest	19.64%	24.24%	18.74%	22.78%
		richest 20%	54.23%	24.17%	30.61%	25.88%
		*Gini/CI*	0.5805^b^	0.1527^b^	0.2036^a^	0.1662^b^
		*(SE)*	(0.0092)	(0.0549)	(0.0732)	(0.0469)
		*KI*	-	−0.4278^a^	−0.3769^a^	−0.4143^a^
		*(SE)*		(0.0559)	(0.0739)	(0.0480)
		Dominance test				
		-against 45^0^ line		D -	D -	D -
		-against Lorenz curve		D +	D +	D +
	Rural (B)	poorest 20%	8.05%	10.76%	7.43%	9.59%
		2nd poorest	12.41%	11.77%	11.03%	11.51%
		3rd	16.45%	19.42%	17.35%	18.69%
		2nd richest	22.11%	30.04%	17.46%	25.64%
		richest 20%	40.98%	28.01%	46.74%	34.56%
		*Gini/CI*	0.3921^b^	0.2664^a^	0.4497^a^	0.3305^b^
		*(SE)*	(0.0041)	(0.0924)	(0.1373)	(0.0778)
		*KI*	-	−0.1257^a^	0.0576^a^	−0.0616^a^
		*(SE)*		(0.0924)	(0.1371)	(0.0777)
		Dominance test				
		-against 45^0^ line		D -	D -	D -
		-against Lorenz curve		none	none	none
2007	Urban (C)	poorest 20%	7.70%	13.36%	8.77%	10.77%
		2nd poorest	11.88%	20.60%	8.72%	13.89%
		3rd	16.06%	14.91%	13.76%	14.26%
		2nd richest	21.86%	21.83%	27.41%	24.98%
		richest 20%	42.51%	29.30%	41.34%	36.10%
		*Gini/CI*	0.4154^b^	0.1581^a^	0.4433^a^	0.3193^b^
		*(SE)*	(0.0049)	(0.0513)	(0.0789)	(0.0513)
		*KI*	-	−0.2572^a^	0.0280^a^	−0.0961^a^
		*(SE)*		(0.0515)	(0.0790)	(0.0514)
		Dominance test				
		-against 45^0^ line		none	D -	D -
		-against Lorenz curve		D +	none	none
	Rural (D)	poorest 20%	9.03%	13.73%	5.36%	8.88%
		2nd poorest	13.31%	16.42%	7.68%	11.36%
		3rd	17.07%	18.57%	16.97%	17.65%
		2nd richest	22.10%	22.40%	20.95%	21.56%
		richest 20%	38.49%	28.88%	49.04%	40.55%
		*Gini/CI*	0.3507^b^	0.2006^a^	0.5375^a^	0.3957^b^
		*(SE)*	(0.0032)	(0.0506)	(0.0652)	(0.0449)
		*KI*	-	−0.1501^a^	0.1868^a^	0.0450^a^
		*(SE)*		(0.0506)	(0.0650)	(0.0447)
		Dominance test				
		-against 45^0^ line		D -	D -	D -
		-against Lorenz curve		none	D -	none
Inequality difference	Δ(Urban–rural)	2002 (A-B)	-	−0.3021	−0.4346	−0.3527
		Dominance test		none	none	none
		2007 (C-D)	-	−0.1071	−0.1589	−0.1411
		Dominance test		none	none	none
	Δ(2007–2002)	Urban (C-A)	-	0.1706	0.4049	0.3182
		Dominance test		none	none	none
		Rural (D-B)	-	−0.0244	0.1292	0.1065
		Dominance test		none	none	none

None indicates failure to reject the null hypothesis that curves are indistinguishable at the 5 percent significance level

D+/D- indicates concentration curve dominates (is dominated by) the Lorenz curve or concentration curve in one year or area and dominates (is dominated by) the other in another year or area

^a^Significant at 0.05

^b^Significant at 0.01

In 2007, the economic gap between the rich and poor narrowed in both urban and rural areas as indicated by the decreasing Gini coefficient. Within a similar economic context, the pro-rich trend of government subsidies for healthcare differed between outpatient and inpatient care in both urban and rural regions. In cities, the CI value increased over this period in both outpatient (0.1527 to 0.1581) and inpatient (0.2036 to 0.4433) care. In rural areas, on the other hand, the CI value of outpatient care decreased (0.2664 to 0.2006) while that of inpatient care increased (0.4497 to 0.5375). In other words, a relatively higher healthcare subsidy was allocated to rural outpatient care in 2007 than in 2002.

**Fig. 2:**
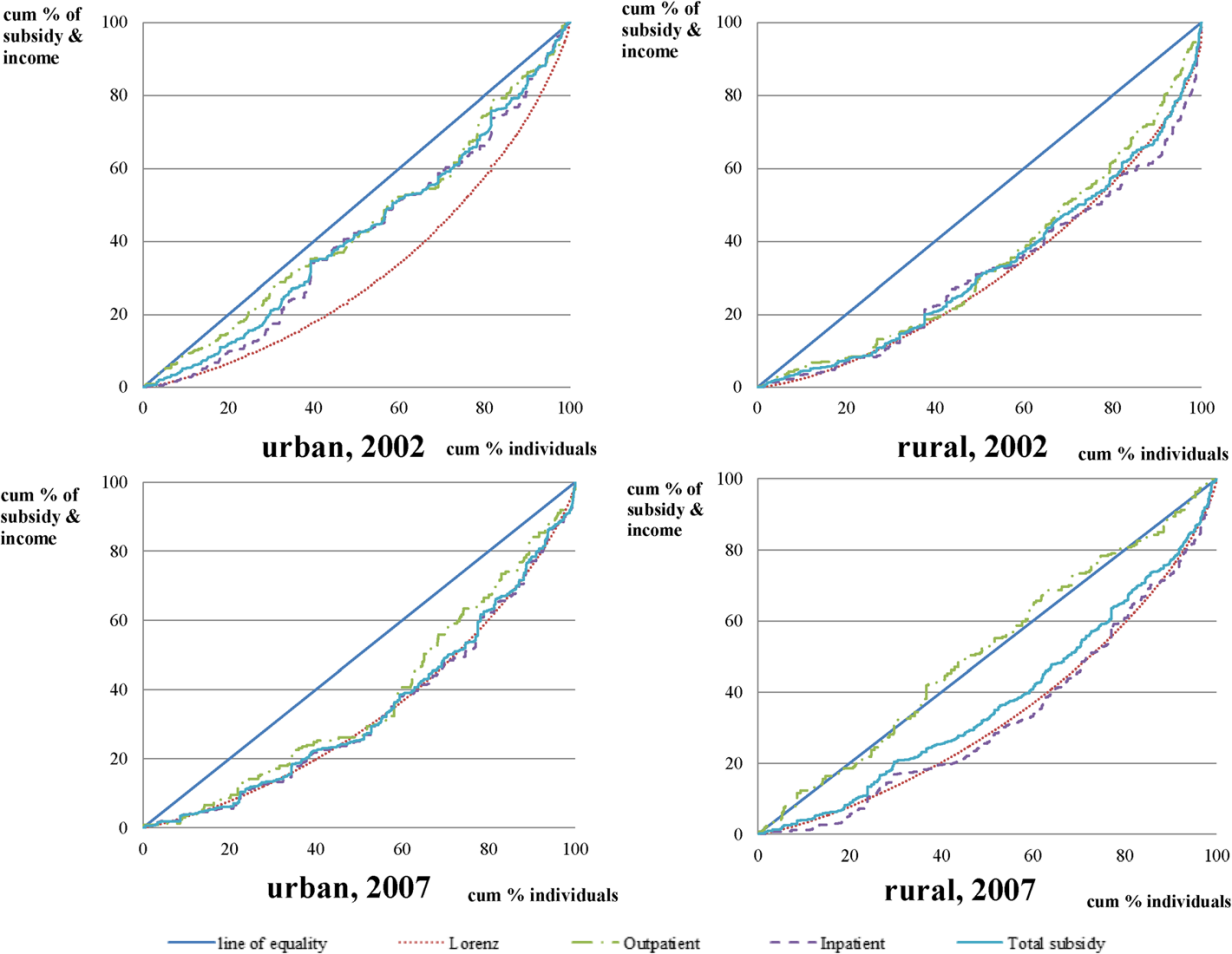
Concentration curve for government subsidies on health care and income. Actual concentration curve for government subsidies on healthcare (including outpatient, inpatient, and total healthcare) and L_2_ in years 2002 and 2007 for both urban and rural areas

All the KIs for outpatient services were negative in 2002 and 2007, indicating that the subsidies to outpatients was distributed progressively to the ATP. With regard to inpatient care, the KI values were all positive except for the case of urban inpatient care in 2002. These results suggest that government subsidies on outpatient care reduced inequality, while the subsidies on inpatient care did not. Results showing the plots of Lorenz curve and the concentration curve are presented in Fig. 2. This provides a visual representation of the progressivity of government subsidies on healthcare.

A comparison between urban and rural areas shows that in both years, 2002 (row A-B) and 2007 (row C-D), the differences in the subsidies were negative (for outpatients −0.3021 and inpatients −0.4346 in 2002, and outpatients −0.1071 and inpatients −0.1589 in 2007). This implies that in terms of the inequality-reducing effect, subsidies to urban healthcare institutes had a better performance than that in rural institutes in both years.

In addition, a comparison of the differences between the subsidies in 2002 and 2007 showed that in the urban areas (row C-A), the KI values were positive for both outpatients (0.1706) and for inpatients (0.4049), while in the rural areas (row D-B) it was negative for outpatients (−0.0244) and positive for inpatients (0.1292). This finding suggests that the inequality-reducing effect had worsened between 2002 and 2007 in inpatient services and urban outpatient services. While for outpatient services in rural areas, the relative gap had decreased.

## Discussion

This study has determined that Chinese government subsidies on healthcare have been equitably distributed in outpatient services but “not yet” to the inpatient services. All GHS KIs for inpatient services were positive in both urban and rural areas in 2007. This indicates that the government health subsidies were concentrated on the rich relative to their ATPs. On the other hand, GHS KIs for outpatient services were all negative in 2007, suggesting the GHS was pro-poor after adjusting for the ATP. Additionally, it has been demonstrated that the equity of GHS has improved from 2002 to 2007 in outpatient services in rural areas but not in outpatient services in urban areas nor all inpatient services in both rural and urban areas. Equity in GHS on inpatients services was not improved based on the increased KI values both in urban (KI increased by 0.4049 from 2002 to 2007) and rural areas (KI increased by 0.1292). However, in rural outpatient care, GHS was inequality-reducing as indicated by the decreased KI value (KI decreased by 0.0244 from 2002 to 2007).

In 2002, the proportion of patients that used the insurance scheme was on a low level in both cities and villages. In addition, health insurance coverage in villages was lower than in cities in 2002, whereas by 2007 the coverage had become much higher than in the urban areas (Table 1). The records kept by NCMS showed that the participation rate was similar amongst the income quintiles in 2007, on the contrary, the participation rate of the urban health insurance increased with the level of income. This difference can largely be attributed to the insurance policy entitlement of UWBMI, which is the major part of urban health insurance. Only citizens with stable jobs were eligible to be insured by UWBMI, although after 2007, migrant workers and other informal workers were allowed to be insured. As a result, the UWBMI rate was strongly associated with income status and by the variation in the proportion of patients receiving reimbursement.

From 2002 to 2007, the proportion of rural residents who used health insurance increased in all income quintiles, whilst the proportion only increased in the middle- and high-income quintiles in urban areas (Table 1). Consequently, the expanding coverage and the proportion requiring compensation, improved the access to and utilization of the healthcare, resulting in an increased chance to receive government subsidies, especially for the poor and vulnerable groups in rural areas. This divergence of the equity of the government health subsidies between urban and rural areas could be explained by the ‘law of inverse equity’, in which the rich receive more of the benefits of publicly provided services when coverage is low, but as coverage increases, the poor start benefiting equally [16].

The difference in GHS in urban and rural areas may be explained by the difference in insurance coverage and in the proportion of patients receiving compensation. However, we found that KI for inpatient care increased from 2002 to 2007 in rural areas while for outpatient care it decreased over the same period. A possible explanation for the difference observed between outpatient and inpatient care in villages and why the equity for government health subsidy was exclusively improved in outpatient care in rural areas, follows.

The divergence of equity between outpatient and inpatient care might be attributed to the compensation policy and benefit package of NCMS. In order to achieve UHC, an important policy goal of China’s healthcare reform, it is significant that every Chinese, irrespective of social standing, not only has access to basic medical care but also gets financial protection. Thus, a key objective of NCMS is to broaden coverage for more rural resident and ensure breadth of UHC. However, other key components of UHC, such as the depth and height of health insurance cover, appear to have received little attention. Since the funding of NCMS was administered and implemented at the county level, fragmentation occurred which limited the scope for risk pooling and made financial protection underprovided. In addition, county officials who were financially responsible for safely managing the funds had to decide between updating benefit packages or expanding population coverage. Based on the policy priority of covering more of the population, NCMS was designed for comprehensive population coverage at the expense of appropriate financial protection.

Although NCMS deductibles were not so high that the insured could not get reimbursed, the ceiling was also very low. Additionally, the cost sharing and fees were very high for rural patients [17, 18]. Also, the insurance scheme indicates that high-expenditure diseases were compensated to a very low level of reimbursement, while low-medical-expenditure ailments, which usually require outpatient services, were compensated to a relatively high level of reimbursement. Involvement in the scheme was directly related to the level of OOP payment. OOP expenditures for rural outpatient care were decreased by a significant magnitude between 2002–2007, whilst other types of OOP payments were increased and remained at a high level (Table 1). Healthcare utilization for the rural population will increase with lower user fees, and the poor who need medical services will begin to benefit from government health spending. For these reasons, it was exclusively the rural outpatient services that improved as a result of equity of government health subsidies.

Another difficulty faced with the government health scheme was due to the financial burdens faced by patients. This burden affected patients’ responsiveness to the care offered as outpatient or inpatient, and influenced their behavior in seeking care and in making hospital-related choices [19, 20]. Their response was not made according to the healthcare need, but by the compensation policy of the insurance scheme. As a result, the reimbursement policies of NCMS have changed in an attempt to encourage individuals, especially those poor who need hospitalization care, to seek outpatient care as a replacement therapy. For example, many rural patients treated illness with Chinese traditional medicine as prescribed in outpatient services. These accounted for as much as 40% of all healthcare delivered in China [21]. For this reason, benefit distribution for rural outpatient care was increased to the poor.

Our study has several limitations that should be addressed. First, patients that have the same ATP would be assigned to the different fractional ranks, thus CI estimates might be unstable and inconsistent [22]. In our study, the patients that have the same ATP were ranked according to their alphabetic order and household number, but a stata module, CONCINDC, is a better method to calculate consistent and replicable estimates of CI [23]. Second, the classification of living standards was based on the household expenditure. Despite that expenditure has been recognized as a preferred measure of living standard [10], self-reported household expenditure might be inaccurate due to recall bias. Consequently, it should be acknowledged that our study was limited to the use of self-reported household expenditure to classify living standards.

## Conclusions

Unlike health financing equity, BIA focuses on whether or not health resources allocated by authorities were evenly distributed among income groups. The present study reflects the overall picture of unequal government health subsidies. With the exception of rural outpatient care in 2007, greater subsidies on various types of healthcare were concentrated on the rich and did not show the inequality-reducing effect in different regions in the years that were studied. However, equity of the rural outpatient benefit was partly driven by the fact that some people were not able to afford hospital expenses and had to choose outpatient care. This patient care-seeking behavior was strongly influenced by China’s current health insurance scheme. With more than 96% of the population having been covered by health insurances, it is therefore indicated that authorities should redesign the present insurance schemes, especially the compensation policies, to account for patient’s actual needs, both in outpatient and inpatient care.

## Abbreviations

ATP: Ability-to-pay
BIA: Benefit incidence analysis
CI: Concentration index
CMS: Rural Cooperative Medical Scheme
GHS: Government Healthcare Subsidies
KI: Kakwani index
NCMS: New Rural Cooperative Medical Scheme
NHSS: National Health Services Survey
OOP: Out-of-pocket payment
UHC: Universal Health Coverage
URBMI: Urban Resident Basic Medical Insurance
UWBMI: Urban Workers Basic Medical Insurance
